# Supplementary material for: Shotgun metagenomic analysis of the oral microbiomes of children with noma
Source: PLoS Negl Trop Dis. 2026 Mar 20;20(3):e0014118. doi: 10.1371/journal.pntd.0014118 (PMC13029773; doi:10.1371/journal.pntd.0014118)
Supplement: S1 Table — (DOCX) [file pntd.0014118.s001.docx]

**S1_Table. Symptoms, Clinical Assessments, and Treatment**

|  | **N = 19*^1^*** | **%** |
| --- | --- | --- |
| **How many days ago did your child fall sick?** | 7 (4, 14) |  |
| **How many days ago did your child fall sick - group?** |  |  |
| <2 | 0 | 0.0% |
| 2 to 6 | 7 | 36.8% |
| 7 to 14 | 8 | 42.1% |
| 15 and above | 4 | 21.1% |
| **What were the first signs and symptoms you noticed** |  |  |
| Painful mouth | 18 | 94.7% |
| Foul mouth smelling | 14 | 73.7% |
| Red gum | 11 | 57.9% |
| Sore gums | 10 | 52.6% |
| Swollen cheek | 10 | 52.6% |
| Bleeding gums | 8 | 42.1% |
| Hole in cheek | 6 | 31.6% |
| **Visible bleeding at the time of sampling** | 6 | 31.6% |
| **Noma stage on admission** |  |  |
| Stage 1: Gingivitis | 10 | 5.3% |
| Stage 2: Oedema | 9 | 47.4% |
| Stage 3: Gangrenous stage | 7 | 36.8% |
| Stage 4: Scarring stage | 2 | 10.5% |
| Weight | 11.5 [9.4, 14.0] |  |
| Height | 89.3 [82.5, 97.0] |  |
| **Clinical assessments** |  |  |
| Noma (acute, with active infection) | 19 | 100.0% |
| **Visit outcome** |  |  |
| Admission | 18 | 94.7% |
| Outparticipant | 1 | 5.3% |
| **Treatment information** |  |  |
| Antibiotics + wound dressing | 17 | 89.5% |
| Admitted for physiotherapy | 1 | 5.3% |
| Antibiotics (outparticipant) | 1 | 5.3% |
| **MUAC (under 5 years)** | **N = 13** |  |
| >=12.5 | 9 | 69.2% |
| 11.5 to 12.4 | 2 | 15.4% |
| <11.5 | 2 | 15.4% |
| **BMI (5years and above)** | **N = 5** |  |
| Healthy weight | 3 | 60.0% |
| Under weight | 2 | 40.0% |
| *^1^* Median (IQR); n (%) |  |  |
