# Supplementary material for: Shotgun metagenomic analysis of the oral microbiomes of children with noma
Source: PLoS Negl Trop Dis. 2026 Mar 20;20(3):e0014118. doi: 10.1371/journal.pntd.0014118 (PMC13029773; doi:10.1371/journal.pntd.0014118)
Supplement: S2 Table — (DOCX) [file pntd.0014118.s002.docx]

**S2_Table. Participant Demographic Information.**

|  | **N = 19***^1^* | **%** |
| --- | --- | --- |
| **Age group of participants** |  |  |
| 2 to 6 | 16 | 84.2% |
| 7 and above | 3 | 15.8% |
| **Sex** |  |  |
| Male | 10 | 52.6% |
| Female | 9 | 47.4% |
| **Education** |  |  |
| Undercare | 13 | 68.4% |
| Arabic school | 6 | 31.6% |
| **Tribe** |  |  |
| Hausa | 19 | 100.0% |
| **State** |  |  |
| Sokoto | 9 | 47.4% |
| Zamfara | 6 | 31.6% |
| Kebbi | 2 | 10.5% |
| Kaduna | 1 | 5.3% |
| Kano | 1 | 5.3% |
