# Supplementary material for: Shotgun metagenomic analysis of the oral microbiomes of children with noma
Source: PLoS Negl Trop Dis. 2026 Mar 20;20(3):e0014118. doi: 10.1371/journal.pntd.0014118 (PMC13029773; doi:10.1371/journal.pntd.0014118)
Supplement: S3 Table — (DOCX) [file pntd.0014118.s003.docx]

**S3_Table. Health Facility Visit and Treatment**

|  | **N = 19***^1^* | % |
| --- | --- | --- |
| **Has your child visited an oral health care provider for a check-up in the past year?** |  |  |
| Yes | 2 | 10.5% |
| No | 18 | 89.5% |
| **If yes, where was this care provided?** |  |  |
| PHC | 2 | 100.0% |
| **Is this the first healthcare facility you visited for your child's current illness?** |  |  |
| Yes | 15 | 78.9% |
| No | 4 | 22.9% |
| **If no, which health facility/provider did you visit before you came here?** |  |  |
| PHC | 3 | 75.0% |
| Chemist/ Drugstore | 1 | 25.0% |
| **Did you face any challenges seeking healthcare at the Noma Children's Hospital?** |  |  |
| Yes | 2 | 11.1% |
| No | 17 | 88.9% |
| **If yes, what challenges did you face when seeking healthcare at the Noma Children's Hospital?** |  |  |
| Expensive transportation | 2 | 100.0% |
