# Supplementary material for: Shotgun metagenomic analysis of the oral microbiomes of children with noma
Source: PLoS Negl Trop Dis. 2026 Mar 20;20(3):e0014118. doi: 10.1371/journal.pntd.0014118 (PMC13029773; doi:10.1371/journal.pntd.0014118)
Supplement: S4 Table — (DOCX) [file pntd.0014118.s004.docx]

**S4_Table:** **sequencing and assembly statistics**

| Participant | Sample | Total read pairs | Read pairs after QC (trimming and host removal) | Assembly size (contigs ≥ 500 bp) | N50 (contigs ≥ 500 bp) | Number of high-quality MAGs after dereplication by participant (accessions) |
| --- | --- | --- | --- | --- | --- | --- |
| N1 | A1 (saliva) | 64281112 | 16893748 | 254629531 | 2142 | 11 (SAMN49825454 – SAMN49825464) |
| N2 | A2 (saliva) | 50928353 | 16336392 | 184056468 | 3062 | 14 (SAMN49825465 – SAMN49825478) |
| N3 | A3 (saliva) | 45743028 | 4241479 | 78061726 | 1320 | 1 (SAMN49825479) |
| N4 | A4 (saliva) | 47927475 | 8321837 | 129304994 | 1875 | 5 (SAMN49825480 – SAMN49825484) |
|  | A24 (swab) | 123536877 | 75562923 | 333554760 | 3711 | 8 (SAMN49825513 – SAMN49825520) |
| N5 | A5 (saliva) | 41944770 | 4587672 | 86746604 | 1752 | 2 (SAMN49825485 – SAMN49825486) |
|  | A25 (swab) | 44269414 | 5265017 | 83423090 | 1846 | 4 (SAMN49825521 – SAMN49825524) |
| N6 | A6 (saliva) | 57102017 | 17213327 | 160491827 | 3109 | 11 (SAMN49825487 – SAMN49825497) |
|  | A26 (swab) | 55758616 | 39105608 | 328641902 | 4973 | 14 (SAMN49825525 – SAMN49825538) |
| N7 | A7 (saliva) | 51427967 | 4071728 | 63197809 | 1379 | 1 (SAMN49825498) |
|  | A27 (swab) | 80359516 | 12230736 | 166227595 | 2380 | 6 (SAMN49825539 – SAMN49825544) |
| N8 | A9 (saliva) | 49595044 | 5577862 | 77502193 | 15700 | 0 |
| N9 | A8 (saliva) | 54492396 | 5554498 | 44563049 | 2273 | 0 |
|  | A28 (swab) | 73418713 | 5162974 | 43487141 | 2698 | 3 (SAMN49825545 – SAMN49825547) |
| N10 | A10 (saliva) | 47935661 | 5996427 | 79302390 | 2248 | 6 (SAMN49825499 – SAMN49825504) |
|  | A30 (swab) | 53954624 | 20244250 | 212023085 | 3724 | 12 (SAMN49825548 – SAMN49825559) |
| N11 | A11 (saliva) | 55300443 | 4503881 | 62806768 | 1077 | 1 (SAMN49825505) |
| N12 | A13 (saliva) | 53584118 | 9845598 | 147013449 | 2971 | 4 (SAMN49825506 – SAMN49825509) |
| N13 | A14 (saliva) | 43109909 | 3229142 | 48000936 | 1199 | 1 (SAMN49825510) |
| N13 | A34 (swab) | 52469528 | 7720060 | 93275768 | 3271 | 8 (SAMN49825560 – SAMN49825567) |
| N14 | A35 (swab) | 60088001 | 4943757 | 57169435 | 1517 | 1 (SAMN49825568) |
| N15 | A17 (saliva) | 48494071 | 2763988 | 30964140 | 1399 | 2 (SAMN49825511 – SAMN49825512) |
| N16 | A16 (saliva) | 60803879 | 4468262 | 64176859 | 1060 | 0 |
| N17 | A18 (saliva) | 47028706 | 4066316 | 50244746 | 1084 | 0 |
|  | A38 (swab) | 40226874 | 5260157 | 89018202 | 1707 | 0 |
| N18 | A19 (saliva) | 43212670 | 4079958 | 32462753 | 1388 | 0 |
|  | A39 (swab) | 48791396 | 3961997 | 56271887 | 1936 | 5 (SAMN49825569 – SAMN49825573) |
| N19 | A40 (saliva) | 52551562 | 47111124 | 204316507 | 6085 | 19 (SAMN49825574 – SAMN49825592) |
