# Supplementary material for: Shotgun metagenomic analysis of the oral microbiomes of children with noma
Source: PLoS Negl Trop Dis. 2026 Mar 20;20(3):e0014118. doi: 10.1371/journal.pntd.0014118 (PMC13029773; doi:10.1371/journal.pntd.0014118)
Supplement: S5 Table — (DOCX) [file pntd.0014118.s005.docx]

**S5_Table. Identification of *Treponema sp*. A in noma participant oral microbiome samples in MAGs and 16S rRNA genes.**

| **Noma disease stage** | **Participant** | **Sample** | ***Treponema sp*. A medium- to high-quality MAG recovered** | **Partial 16S rRNA gene (min. 500 bp) reconstructed with >99% nucleotide ID to AM420013** | ***Treponema sp*. A identified** |
| --- | --- | --- | --- | --- | --- |
| **1** (gingivitis) | N17 | A18 (saliva) | No | No | **No** |
|  |  | A38 (swab) | No | No |  |
| **2**  (oedema) | N1 | A1 (saliva) | Yes (HQ) | Yes (1431 bp) | **Yes** |
|  | N3 | A3 (saliva) | No | Yes (1430 bp) | **Yes** |
|  | N4 | A4 (saliva) | No | No | **No** |
|  |  | A24 (swab) | No | No |  |
|  | N5 | A5 (saliva) | Yes (HQ) | Yes (1294 bp) | **Yes** |
|  |  | A25 (swab) | Yes (HQ) | Yes (1292 bp) |  |
|  | N6 | A6 (saliva) | No | Yes (1524 bp) | **Yes** |
|  |  | A26 (swab) | Yes (MQ) | Yes (1531 bp) |  |
|  | N9 | A8 (saliva) | Yes (HQ) | Yes (1466 bp) | **Yes** |
|  |  | A28 (swab) | Yes (HQ) | Yes (1475 bp) |  |
|  | N10 | A10 (saliva) | No | Yes (678 bp) | **Yes** |
|  |  | A30 (swab) | Yes (MQ) | Yes (1537 bp) |  |
|  | N13 | A14 (saliva) | Yes (MQ) | Yes (1444 bp) | **Yes** |
|  |  | A34 (swab) | Yes (HQ) | Yes (1426 bp) |  |
|  | N18 | A19 (saliva) | No | No | **Yes** |
|  |  | A39 (swab) | No | Yes (843 bp) |  |
| **3**  (gangrene) | N2 | A2 (saliva) | Yes (HQ) | Yes (1493 bp) | **Yes** |
|  | N7 | A7 (saliva) | No | No | **Yes** |
|  |  | A27 (swab) | No | Yes (698 bp) |  |
|  | N8 | A9 (saliva) | No | Yes (541 bp) | **Yes** |
|  | N12 | A13 (saliva) | Yes (HQ) | Yes (1331 bp) | **Yes** |
|  | N14 | A35 (swab) | Yes (MQ) | Yes (1155 bp) | **Yes** |
|  | N15 | A17 (saliva) | Yes (HQ) | Yes (1412 bp) | **Yes** |
|  | N19 | A40 (swab) | Yes (HQ) | Yes (1101 bp) | **Yes** |
| **4**  (scarring) | N11 | A11 (saliva) | No | No | **No** |
|  | N16 | A16 (saliva) | No | No | **No** |
