# Supplementary material for: Shotgun metagenomic analysis of the oral microbiomes of children with noma
Source: PLoS Negl Trop Dis. 2026 Mar 20;20(3):e0014118. doi: 10.1371/journal.pntd.0014118 (PMC13029773; doi:10.1371/journal.pntd.0014118)
Supplement: S1 Fig — (DOCX) [file pntd.0014118.s007.docx]

**S1_Fig: counts of *Treponema* across noma stages**
Violin plots show the normalised counts of *Treponema* across noma stages (1 – 4).

**
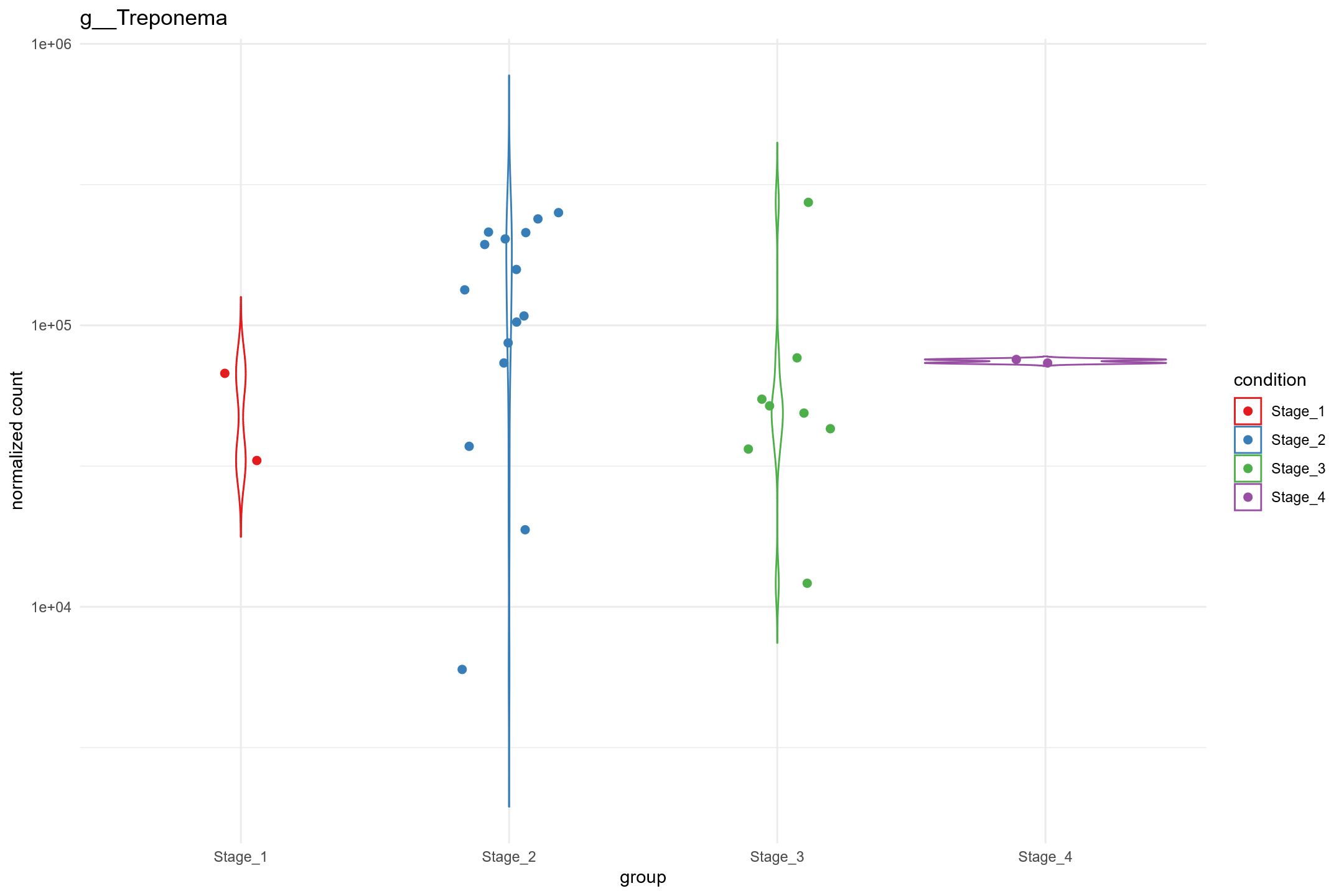
**
