# Supplementary material for: Shotgun metagenomic analysis of the oral microbiomes of children with noma
Source: PLoS Negl Trop Dis. 2026 Mar 20;20(3):e0014118. doi: 10.1371/journal.pntd.0014118 (PMC13029773; doi:10.1371/journal.pntd.0014118)
Supplement: S2 Fig — (DOCX) [file pntd.0014118.s008.docx]

**S2 Fig. Counts of twelve genera across the noma dataset and the three separate healthy cohorts.**


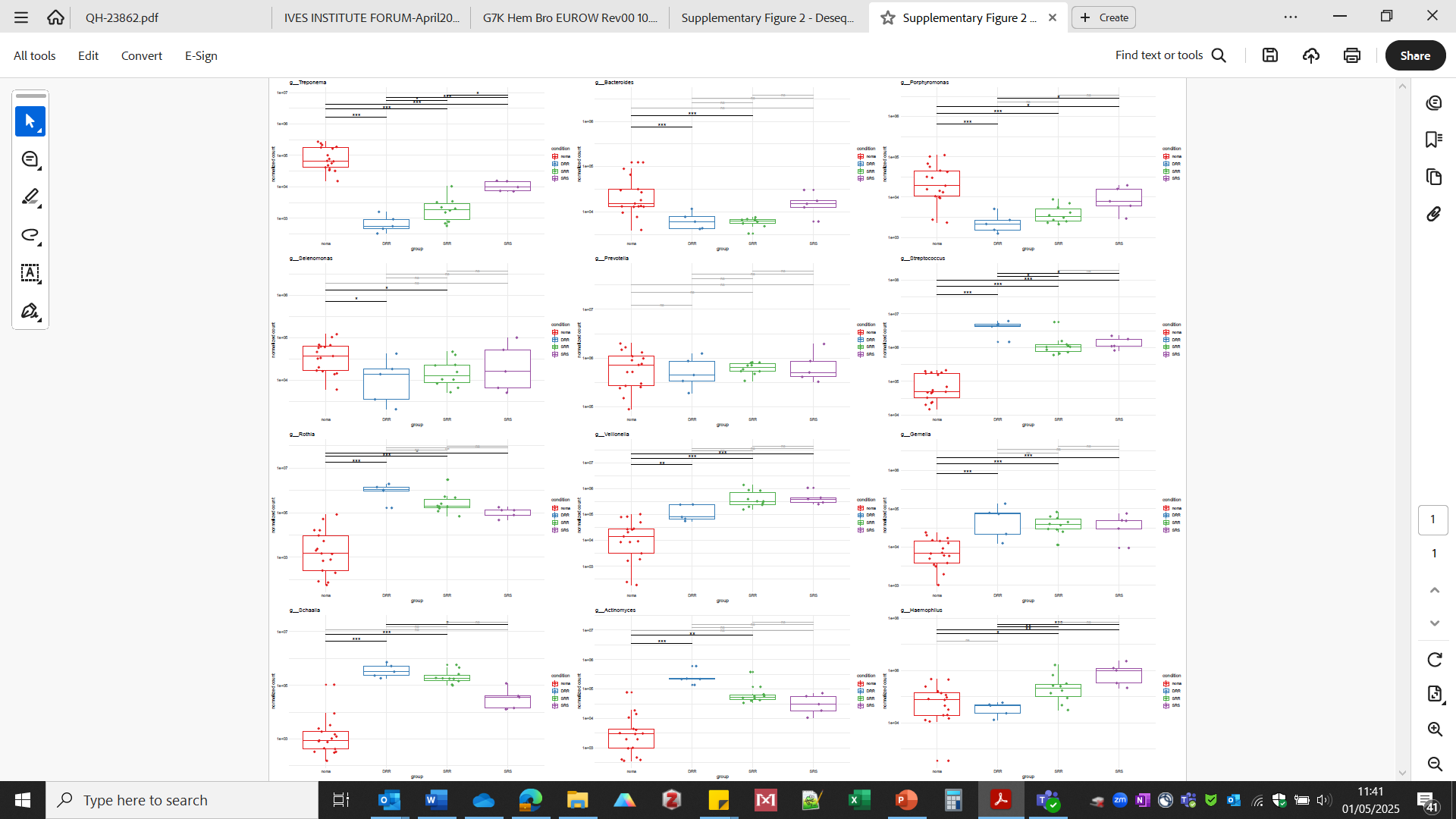


Boxplots show the normalised counts of 12 genera in the noma dataset and the three separate healthy datasets. The healthy datasets are named according to their accession prefix i.e. DRR, SRR, SRS. As detailed in Table S5, DRR were from Japan, SRR from Denmark and SRS from the USA. Significance was determined using the DESeq2 R package. Pairwise Wald tests were performed between all healthy datasets and the noma dataset. The p-values displayed are adjusted p-values calculated using Benjamini-Hochberg correction. Significance thresholds: *p* < 0.05; **p* < 0.01; ***p* < 0.001; “ns” = not significant.
