# Supplementary material for: Shotgun metagenomic analysis of the oral microbiomes of children with noma
Source: PLoS Negl Trop Dis. 2026 Mar 20;20(3):e0014118. doi: 10.1371/journal.pntd.0014118 (PMC13029773; doi:10.1371/journal.pntd.0014118)
Supplement: S3 Fig — (DOCX) [file pntd.0014118.s009.docx]

**S3_Fig. Statistical differences in relative abundance of genera in healthy control and noma samples of top 20 genera.**


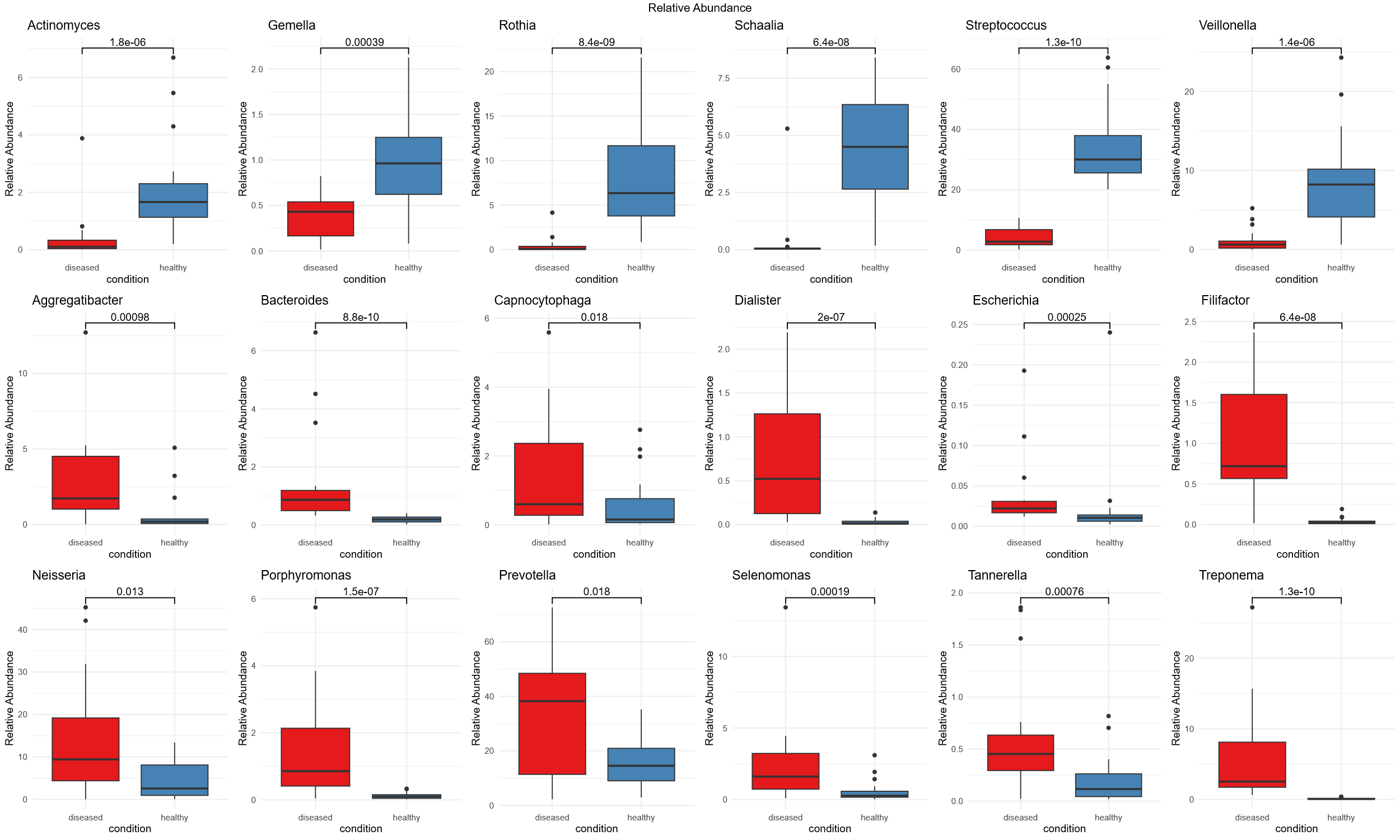


For *Escherichia* sample A8 was removed as an outlier. Statistical significance was calculated using the wilcox test.
