# Supplementary figures and images for: Shotgun metagenomic analysis of the oral microbiomes of children with noma
Source: PLoS Negl Trop Dis. 2026 Mar 20;20(3):e0014118. doi: 10.1371/journal.pntd.0014118 (PMC13029773; doi:10.1371/journal.pntd.0014118)

**S4_Fig. Top 1% of genera across entire dataset.**

**
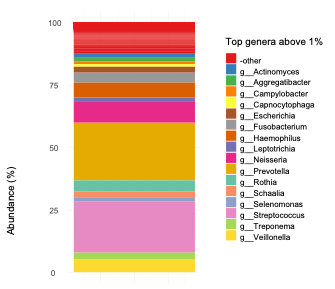
**

Supplement: S4 Fig — (DOCX) [file pntd.0014118.s010.docx]
