# Supplementary material for: Shotgun metagenomic analysis of the oral microbiomes of children with noma
Source: PLoS Negl Trop Dis. 2026 Mar 20;20(3):e0014118. doi: 10.1371/journal.pntd.0014118 (PMC13029773; doi:10.1371/journal.pntd.0014118)
Supplement: Attachment — Submitted filename: Review report.docx [file pntd.0014118.s012.docx]

Noma is a necrotizing disease that primarily affects malnourished and immunocompromised children. It is believed to result from a polymicrobial infection, and no single definitive causative agent has been identified to date. The manuscript by Olaleye et al. presents compelling and novel findings that suggest a probable causative organism—Treponema sp. A—identified from the complex oral microbiome of children at various stages of Noma. The authors also provide detailed information regarding sample inclusion/exclusion criteria, sample handling, storage, and analytical methods. However, the study relies heavily on metagenomic analysis using shotgun sequencing, a technique that carries inherent limitations and biases. To strengthen the impact and accuracy of the findings, further validation through isolation and culture of Treponema sp. A along with in vitro infection experiments would be ideal. Additionally, the relatively small sample size (across different Noma stages, age ranges, and genders) limits the strength of the conclusions.I have a few minor comments:

1. Lines 62–64: The sentence is unclear and should be rewritten for clarity.
2. Figure 1A & 1B: Fusobacterium abundance appears higher compared to the global dataset, yet this difference is not reflected in other analyses (Fig. 2). Please include differential abundance data for Fusobacterium in Figure 2.
3. Lines 403–413: Several analyses are referenced, but the corresponding table or figure is missing.
4. Please apply the Oxford comma consistently throughout the manuscript.
5. Lines 506–508: This sentence is confusing and should be rephrased for clarity.
6. The Discussion section is lengthy and contains extensive literature review, which distracts from the main findings. Removing unnecessary background information would improve the focus and readability.
7. Line 652: Please include the name of the company providing the kit.
