# Supplementary material for: Shotgun metagenomic analysis of the oral microbiomes of children with noma
Source: PLoS Negl Trop Dis. 2026 Mar 20;20(3):e0014118. doi: 10.1371/journal.pntd.0014118 (PMC13029773; doi:10.1371/journal.pntd.0014118)
Supplement: Attachment — Submitted filename: response to reviewer comments.docx [file pntd.0014118.s014.docx]

Dear Editor,

Thank you for providing the opportunity for us to submit a revised version of our manuscript. We thank the reviewers for their time, comments and positive review of our study.

Below are point-by-point responses (in red) to editorial requirements and reviewer comments.

Yours sincerely

Stuart Ainsworth

1) Please ensure that the CRediT author contributions listed for every co-author are completed accurately and in full.At this stage, the following Authors/Authors require contributions: Michael Olaleye, Angus M. O'Ferrall, Richard N. Goodman, Deogracia Wa Kabila, Miriam Peters, Gregoire Falq, Joseph Samuel, Donal Doyle, Diana Gomez, Gbemisola Oloruntuyi, Shafi'u Isah, Adeniyi Semiyu Adetunji, Elise Farley, Nichoas J Evans, Mark Sherlock, Adam P Roberts, Mohana Amirtharajah, and Stuart Robert Ainsworth. Please ensure that the full contributions of each author are acknowledged in the "Add/Edit/Remove Authors" section of our submission form.
The list of CRediT author contributions may be found here: <https://journals.plos.org/plosntds/s/authorship#loc-author-contributions>

This omission has now been rectified.

2) Please provide an Author Summary. This should appear in your manuscript between the Abstract (if applicable) and the Introduction, and should be 150-200 words long. The aim should be to make your findings accessible to a wide audience that includes both scientists and non-scientists. Sample summaries can be found on our website under Submission Guidelines:
<https://journals.plos.org/plosntds/s/submission-guidelines#loc-parts-of-a-submission>

We have now provided an author summary.

3) Please upload all main figures as separate Figure files in .tif or .eps format. For more information about how to convert and format your figure files please see our guidelines: 
<https://journals.plos.org/plosntds/s/figures>

Complete.

4) We have noticed that you have uploaded Supporting Information files, but you have not included a list of legends. Please add a full list of legends for your Supporting Information files after the references list.

Added.

5) Please amend your detailed Financial Disclosure statement. This is published with the article. It must therefore be completed in full sentences and contain the exact wording you wish to be published.

We have added the following statement “Médecins Sans Frontières provided funding and contributed to study design, data collection, and interpretation of the findings. MSF-affiliated co-authors (M.O., D.W.K., M.P., G.F., J.S., D.D., G.O., E.F., M.S., and M.A.) participated in data analysis and manuscript preparation. The decision to publish was made jointly by the author group.”

1) State the initials, alongside each funding source, of each author to receive each grant. For example: "This work was supported by the National Institutes of Health (####### to AM; ###### to CJ) and the National Science Foundation (###### to AM)."

See Financial Disclosure statement, above. – no specific funding in this case.

2) State what role the funders took in the study. If the funders had no role in your study, please state: "The funders had no role in study design, data collection and analysis, decision to publish, or preparation of the manuscript."

See Financial Disclosure statement, above.

3) If any authors received a salary from any of your funders, please state which authors and which funders.

See Financial Disclosure statement, above.

**Reviewers' Comments:**

**Methods**

Reviewer #1: The global healthy saliva dataset has been used as the comparator but the differences in geography, diet, socio-economic status, oral hygiene, and environmental exposures may confound interpretation. Please mention this methods. We discuss how the data set is obtained in the methods and its inherent limitation in the discussion (starting line 592), so we feel this is already adequately addressed.

Reviewer #2: -Yes, objectives were clearly articulated.
-Yes, study design is appropriate, however, could be better by supplementing bacterial culture and in vitro infection study. We agree with the reviewer. Unfortunately, cultivation of bacteria and infection models (the latter which, as far as we are aware, do not exist for noma) were not within the scope of the study. Fortunately, we have secured further funding and will be performing cultivation studies in a new study starting in the near future.

**Results**
Reviewer #1: The functional analysis from metagenomics data (e.g. metabolic pathways or virulence factors) would have strengthen the study. Functional analysis would ultimately increase the strength of the study, however, due to the number of genomes obtained, this would be a significant undertaking. We are actively investigating this topic.

Results related to stage-specific microbial patterns are limited and sometimes difficult to interpret due to small numbers. Please see this part. Also, the random forest model shows near-perfect classification (AUC = 1.0), which is striking. While technically valid, this result warrants clearer contextualisation.

We thank the reviewer for highlighting this important point. We agree that an AUC of 1.0 is a striking result that often warrants scrutiny regarding overfitting. However, we believe this result is technically robust and biologically reflective of the extreme nature of noma disease for the following reasons:

- As we show in the rest of the paper, noma represents a major shift in the oral microbiome. As shown in Figure 3D, the PCoA ordination, there is no overlap between the noma and healthy samples, this is an unsupervised machine learning algorithm which still picks up the distinction between the two datasets. Therefore the dysbiosis is so profound that the random forests model is easily distinguishing between them.
- To ensure the AUC = 1 result was not a statistical artefact, we ran a permutation test (n = 1000, Figure 3C). This showed that when the labels were shuffled, accuracy dropped significantly (with the histogram showing they were all mostly below 1 hence the p value of 0.022). This shows the AUC of 1.0 was driven by a true biological signal rather than by random chance or the model memorising the data.
- As we mention in our discussion, the lack of local controls from Nigeria is probably contributing to the distinctness of separation. Not only is the model picking up on noma vs healthy but also Nigerian samples vs healthy samples from high-income countries, so together this gives a clean separation represented as an AUC of 1.

We have rewritten the relevant paragraph in the results section to highlight the major dysbiosis underlying the result of AUC = 1. We have added sentences addressing the use of permutation testing to test against the model memorising the data and the limitation that the global healthy dataset introduces more distinction, which further contributes to this clean separation, resulting in an AUC of 1.

**Summary and General Comments**
Reviewer #1: This manuscript presents the first deep shotgun metagenomic analysis of oral microbiomes from children with acute noma. The identification of novel Treponema species such as Treponema sp. A, and the analysis of AMR determinants are notable strengths of the study.

We thank the reviewer for this encouraging comment.

Reviewer #2: Noma is a necrotizing disease that primarily affects malnourished and immunocompromised children. It is believed to result from a polymicrobial infection, and no single definitive causative agent has been identified to date. The manuscript by Olaleye et al. presents compelling and novel findings that suggest a probable causative organism—Treponema sp. A—identified from the complex oral microbiome of children at various stages of Noma. The authors also provide detailed information regarding sample inclusion/exclusion criteria, sample handling, storage, and analytical methods.

We thank the reviewer for this encouraging comment.

However, the study relies heavily on metagenomic analysis using shotgun sequencing, a technique that carries inherent limitations and biases. To strengthen the impact and accuracy of the findings, further validation through isolation and culture of Treponema sp. A along with in vitro infection experiments would be ideal. Additionally, the relatively small sample size (across different Noma stages, age ranges, and genders) limits the strength of the conclusions.

We thank the reviewer and agree that both the metagenomics-only approach and the sample size limit the strength of inference. We have framed the work as hypothesis-generating pilot data and have carefully worded the Results and Discussion to avoid causal interpretation. We also agree that culture and experimental validation of Treponema sp. A would be ideal; however, this is outside the scope of the current manuscript and will be a focus of planned follow-on work.

1. Lines 62–64: The sentence is unclear and should be rewritten for clarity.

This sentence now reads “Noma (cancrum oris) is an orofacial gangrene of unknown aetiology that predominantly affects malnourished, immunocompromised children (most often aged 2–6 years) living in chronic poverty with limited access to healthcare.”

2. Figure 1A & 1B: Fusobacterium abundance appears higher compared to the global dataset, yet this difference is not reflected in other analyses (Fig. 2). Please include differential abundance data for Fusobacterium in Figure 2.

We thank the reviewer for making this suggestion. However, while Figure 1A & 1B show distinction in relative abundance of *Fusobacterium* between noma and healthy samples, Figure 2B-E only plots genera that are significantly (calculated with adjusted p-values) different between the normalised counts in the noma dataset and the global healthy dataset (shown by a star between the boxplots). However, for Fusobacterium, there is not a significant difference in normalised counts between the datasets. Therefore, plotting a boxplot showing normalised counts for Fusobacterium will change the overall meaning of the boxplots in this figure, which is conveying significant differences in normalised counts between the noma and global healthy datasets. We have included a figure below to how the genera already displayed (*Treponema*, *Porphyromonas* etc.) show a significant difference, whereas *Fusobacterium* does not (shown by the lack of star).

3. Lines 403–413: Several analyses are referenced, but the corresponding table or figure is missing.

The underlying data are freely available in the referenced source. We did not include a separate table or figure, as the analysis is described in sufficient detail in the text.

4. Please apply the Oxford comma consistently throughout the manuscript.

We have gone through the manuscript and endeavoured to do this where it seems appropriate.

5. Lines 506–508: This sentence is confusing and should be rephrased for clarity.

We thank the reviewer for highlighting this. We have now changed the text to read “Despite links to oral pathology, polymicrobial disease and conditions that disproportionately affect marginalised populations, the role of *Treponema* and other spirochaetes in noma has often been overlooked, even though they are frequently reported”

6. The Discussion section is lengthy and contains extensive literature review, which distracts from the main findings. Removing unnecessary background information would improve the focus and readability.

We note the comment regarding the length and level of contextual detail in the Discussion. We intentionally include this background because the noma research community is small and multidisciplinary, and many readers are not specialists in microbiology/metagenomics; the additional context is intended to support interpretation of the findings rather than to provide a comprehensive literature review. To address readability, we have made minor edits for clarity and flow.

7. Line 652: Please include the name of the company providing the kit. This in formation has now been added.
